# Impact of oral anaerobic bacteria on the tumor immune microenvironment and prognosis of oral cancer

**DOI:** 10.1186/s12967-025-07189-5

**Published:** 2025-11-12

**Authors:** Kana Kashima, Takuro Saito, Hitomi Kajikawa, Atsunari Kawashima, Azumi Ueyama, Narikazu Uzawa, Hisashi Wada

**Affiliations:** 1 Department of Oral and Maxillofacial Oncology and Surgery, Graduate School of Dentistry, The University of Osaka, Osaka, Japan; 2 Department of Clinical Research in Tumor Immunology, Graduate School of Medicine, The University of Osaka, 2-2 Yamada-Oka, Suita, Osaka, 565-0871 Japan; 3 Department of Gastroenterological Surgery, Graduate School of Medicine, The University of Osaka, Osaka, Japan; 4 Department of Urology, Graduate School of Medicine, The University of Osaka, Osaka, Japan; 5 Drug Discovery and Disease Research Laboratory, Shionogi and Co., Ltd., Osaka, Japan

**Keywords:** Mouth neoplasms, Carcinoma, Squamous cell, Microbiota, Tumor microenvironment, Lymphocytes, Tumor-infiltrating, T Lymphocytes, PD-1 receptor

## Abstract

**Background:**

Oral squamous cell carcinoma (OSCC) accounts for > 90% of oral cancers and has a poor prognosis. The microbiota affects the tumor microenvironment and tumor immune responses; however, the relationship between specific bacterial compositions and tumor-infiltrating immune cells in OSCC remains unclear.

**Methods:**

The microbial diversity and compositions of tumor, normal mucosa, and stool samples from 42 OSCC patients were examined using 16S rDNA sequencing. Bacterial sampling was performed preoperatively by swabbing the tumor surface and normal mucosa and scraping a deep portion of the tumor. Differences in bacterial compositions between samples were examined using a linear discriminant analysis effect size analysis. To investigate the functional states of T cells, tumor-infiltrating immune cells were isolated and subjected to flow cytometry. The relationships among specific bacterial compositions, clinicopathological factors, and tumor-infiltrating immune cells were examined and the potential of microbiota-targeted therapy for OSCC was assessed.

**Results:**

Microbial α-diversity was higher in tumors than in the normal mucosa. Based on the bacterial compositions of tumor surfaces, patients were classified into anaerobic bacteria-dominant Group A (n = 13) and aerobic bacteria-dominant Group B (n = 10). Group A had more advanced cancer stages (*p* = 0.0003), shorter recurrence-free survival (*p* = 0.004), and a higher frequency of exhausted PD-1^+^Tim3^+^CD8^+^ T cells (*p* = 0.01). Four bacterial genera, *Parvimonas, Peptostreptococcus*, *Selenomonas*, and *Streptococcus* were identified in comparisons of tumor surface samples between Groups A and B and between tumor surface and normal surface samples. A scoring system based on the ratio of anaerobic bacteria (*Parvimonas*, *Peptostreptococcus*, and *Selenomonas*) to aerobic bacteria (*Streptococcus*) correlated with impaired immune cell function (*p* = 0.02) and a poor prognosis (HR 9.61, 95% CI 1.15–80.63; *p* = 0.04). A simplified scoring system based on the *Parvimonas* to *Streptococcus* ratio showed a slightly poorer prognosis (HR 2.58, 95% CI 0.50–13.36; *p* = 0.26) and correlated with impaired immune cell function (*p* = 0.03).

**Conclusions:**

This is the first study to show a direct relationship between intratumoral anaerobic bacterial predominance and CD8⁺ T cell exhaustion in OSCC, suggesting a microbiota-dependent mechanism of immune dysfunction and disease progression. The bacteria scoring system has potential as a prognostic marker and guide for microbiota-targeted therapies to enhance anti-tumor immunity.

**Supplementary Information:**

The online version contains supplementary material available at 10.1186/s12967-025-07189-5.

## Background

Cancer remains a major global health concern and despite recent advances in prevention, early detection, and treatment, it continues to be one of the leading causes of mortality worldwide, with an estimated 10 million deaths being reported in 2020 [1, 2]. Among various cancer types, oral cancer, which develops in the tongue, gingiva, buccal mucosa, or floor of the mouth, accounts for approximately 2% of all cancers and 40% of head and neck cancers [1]. Squamous cell carcinoma (SCC) is the most common histological subtype, occurring in > 90% of all cases [3]. Risk factors are smoking, alcohol consumption, chronic irritation, genetic predisposition, periodontitis, poor oral hygiene, and inflammation due to chronic infections [4, 5]. Despite advances in multidisciplinary treatment, including surgery, radiotherapy, and chemotherapy, the 5-year survival rate of oral cancer remains at approximately 50–60% [6]. In recent years, immune checkpoint inhibitor (ICI) therapy has become widespread for various cancers; however, the low response rates of head and neck cancers remain a significant challenge [7, 8]. Therefore, the development of novel therapeutic strategies for oral squamous cell carcinoma (OSCC) is urgently needed.

The relationship between the microbiota and local immune system, which may play a role in the development of systemic diseases, has been attracting increasing attention. In cancer patients, the microbiota is closely associated with the immune cell profile of the tumor microenvironment (TME) [9, 10]. Moreover, the microbiota has been shown to affect the prognosis of cancer patients [11]. In oral cancer, Gram-negative anaerobic bacteria, such as *Fusobacterium* and *Prevotella*, have frequently been detected on the tumor surface, a finding that was confirmed with bacterial cultures [12]. Additionally, 16S rDNA sequencing revealed a significant difference in the bacterial composition between OSCC tumors and the normal oral mucosa [13], with an increased abundance of bacteria associated with periodontitis being detected in OSCC tumors [14]. Therefore, screening of the microbiota has been proposed as a predictive diagnostic tool for oral cancer [15]. Moreover, since tumor-associated bacteria have recently been implicated in the efficacy of cancer immunotherapy [16, 17], the detection of OSCC-specific bacteria may lead to the identification of key regulatory factors that enhance therapeutic responses.

Tumor-infiltrating T cells comprise CD4^+^, CD8^+^ T cells and NK cells, and play a direct and crucial role in antitumor immunity [18]. Tumor antigen-specific activated CD8^+^ T cells, also known as cytotoxic T lymphocytes, have been extensively examined as effector immune cells responsible for tumor elimination. An immunohistochemical (IHC) analysis of fixed tumor tissues showed that CD8^+^ T cells positively correlated with the prognosis of patients with various cancer types [19, 20]. Additionally, a flow cytometric analysis revealed that the status of CD8^+^ tumor-infiltrating lymphocytes (TILs) ranged from young, multifunctional populations (T cell factor 1 (Tcf-1)^+^ Thymocyte selection-associated high mobility group box (TOX)^−^ Programmed death-1 (PD-1)^int^ T-cell immunoglobulin and mucin-domain containing-3 (Tim3)^−^) to highly cytotoxic activated states (PD-1^+^Tim3^−^ Granzyme B (GzmB)^+^IFN-γ^+^), and ultimately to an exhausted state (Tcf-1^−^TOX^+^PD-1^+^Tim3^+^). Tcf-1^+^Tim3^−^CD8^+^ TILs have been shown to correlate with clinical benefits from ICI therapy, whereas PD-1^+^Tim3^+^CD8^+^ TILs are associated with a poor prognosis in patients with renal cell carcinoma [21]. On the other hand, CD4^+^ regulatory T cells (Tregs) suppress antitumor immune responses, and a negative correlation was observed between Tregs and the prognosis of patients with several cancers [19, 20]. Although few studies have investigated the microbiota and immune profiles in OSCC, an increase in CD8^+^ TILs and their distribution, as assessed by IHC, correlated with overall survival (OS) and recurrence-free survival (RFS) [22]. Furthermore, a specific response of CD8^+^ T cells to *Streptococcus salivarius* in peripheral blood mononuclear cells was demonstrated, and the frequency of this response positively correlated with RFS [23]. Although the findings of IHC on tumor tissue and a flow cytometric analysis of peripheral blood in OSCC have been reported, few studies have performed a flow cytometric analysis of TILs purified from fresh tumor tissue [24, 25].

Therefore, the present study examined the relationships among OSCC-specific bacterial compositions, clinicopathological factors, and tumor-infiltrating immune cells and investigated whether interventions targeting the local microbiota and immune environment contribute to improvements in the prognosis of OSCC patients through an analysis of the tumor microbiota.

## Materials and methods

### Patients and bacterial sample collection

**Fig. 1:**
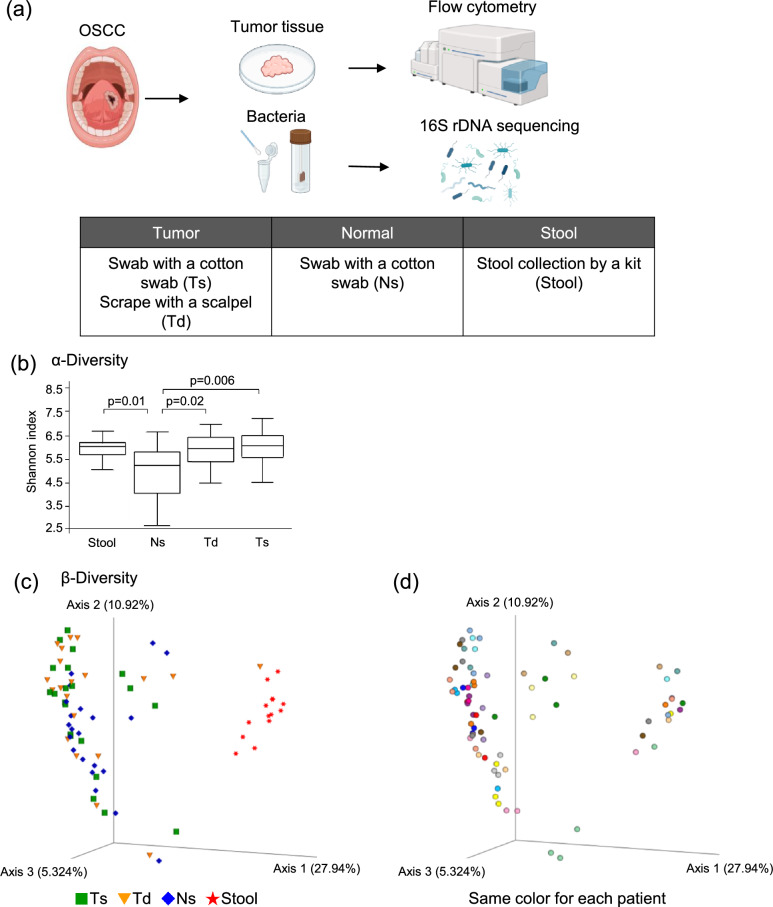
Sampling methods and bacterial diversity analysis. **a** Analytic methods for the microbiota and tumor-infiltrating immune cells. Samples were collected from the tumor surface (Ts), tumor deep tissue (Td), normal mucosal surface (Ns), and stool of each patient. Bacterial compositions were analyzed by 16S rDNA sequencing. Tumor-infiltrating immune cells were extracted from fresh tumor tissue, followed by an analysis using flow cytometry. **b** The α-diversity of the microbiota for each sample was compared using the Shannon index. Green squares represent Ts samples, yellow triangles Td samples, blue squares Ns samples, and red stars stool samples. **c** The β-diversity of the microbiota in each sample was compared using an unweighted UniFrac analysis. Green squares represent Ts samples, yellow triangles Td samples, blue squares Ns samples, and red stars stool samples. **d** The β-diversity of the microbiota in each sample was compared using an unweighted UniFrac analysis. Circles of the same color indicate samples from the same patient

Forty-two primary OSCC patients who underwent curative surgery in our department were included in the present study. As the discovery cohort, 23 patients were enrolled between January 2022 and May 2023, while 19 patients were enrolled as the validation cohort between June 2023 and June 2024. None of the patients analyzed had received preoperative chemotherapy, and all were free from antibiotic use for at least one week prior to sampling. Bacterial samples were collected from the tumor surface (Ts), tumor deep tissue (Td), normal mucosal surface (Ns), and stool of each patient either before surgery or from dissected specimens (Fig. 1a). Samples of Ts and anatomically corresponding Ns on the contralateral side were collected using Sterile Rayon Tipped Applicators (Puritan, USA). Td samples were collected by scraping with a sterile scalpel. Ts, Td, and Ns samples were immediately frozen at –80°C after collection, as previously described [26], to preserve anaerobic bacteria and minimize contamination. Patients abstained from food and drink for at least 12 h before sampling. Stool samples, collected within three days before surgery, were suspended in four times their weight/volume of a 20% glycerol solution in PBS. All samples were stored at − 80°C until analyzed. The present study was approved by the Ethics Committee of the Osaka University Graduate School of Dentistry Hospital (H30-E49-5) and was conducted in accordance with the Declaration of Helsinki. Written informed consent was obtained from all patients.

### DNA extraction and sequencing

DNA was extracted from each sample using the DNeasy PowerSoil Kit (QIAGEN, Hilden, Germany) according to the manufacturer’s protocol. Sequencing of the V1–V2 region was performed based on previously described methods [27]. Amplicons were prepared using a forward primer (16S_27Fmod: TCG TCG GCA GCG TCA GAT GTG TAT AAG AGA CAG AGR GTT TGA TYM TGG CTC AG) and reverse primer (16S_338R: GTC TCG TGG GCT CGG AGA TGT GTA TAA GAG ACA GTG CTG CCT CCC GTA GGA GT). The sequencing library was prepared according to the 16S library preparation protocol provided by Illumina (San Diego, CA, USA). Dual-index adapters for sequencing on the Illumina MiSeq platform were attached using the Nextera XT Index Kit (Illumina, San Diego, CA, USA). Each sequencing library was diluted to a concentration of 5 ng/µL. Equal volumes of the libraries were pooled to a final concentration of 4 nM. The DNA concentration of the pooled library was measured using quantitative PCR (qPCR) with the KAPA SYBR FAST qPCR Master Mix (Roche, Basel, Switzerland) and the primers Primer 1 (AAT GAT ACG GCG ACC ACC) and Primer 2 (CAA GCA GAA GAC GGC ATA CGA).

### Bioinformatics and microbiome analyses

A bioinformatics analysis was performed using QIIME2 as follows: the construction of ASV tables from paired-end sequencing reads. Taxonomic assignment was conducted using the Greengenes database [28] and QIIME2 pipeline (v2020.2) [29]. Data processing and taxonomic assignments were performed through (a) merging, filtering, and denoising paired-end reads using DADA2 [30], and (b) analyzing V1–V2 sequences with Greengenes data for the 16S rDNA V2 region using a QIIME2 naive Bayes classifier to assign taxonomic identities and elucidate bacterial compositions. α-Diversity in sampling sites was evaluated using operational taxonomic unit (OTU)/ASV tables obtained from the QIIME2 analysis at a sequencing depth of 30,000 reads. Differences in diversity between sampling sites were assessed via the Wilcoxon signed-rank test. Bray–Curtis dissimilarities were calculated at the genus level (level 6) using OTU/ASV tables from the V1–V2 region. To evaluate β-diversity among sampling sites for the V1–V2 region, a permutational multivariate analysis of variance was performed and p-values were calculated using the Wilcoxon signed-rank test.

### Sampling of TILs

Fresh tumor tissues resected during surgery were collected and finely minced using surgical scissors. To purify tumor-infiltrating T cells, the Human Tumor Dissociation Kit (Miltenyi Biotec, Bergisch Gladbach, Germany) and gentleMACS Dissociator (Miltenyi Biotec) were used according to the manufacturer’s protocols. TIL samples were available for 20 of 23 patients in the discovery cohort and 15 of 19 patients in the validation cohort due to sampling difficulties or sample quality issues.

### Multicolor flow cytometric analysis

Cells were stained with fixable viability dye (Thermo Fisher Scientific, Waltham, MA, USA) to exclude dead cells and were blocked with Fc receptor blocking solution (BioLegend, San Diego, CA, USA). Cells were then stained with fluorescent dye-conjugated antibodies. The antibody cocktail for flow cytometry included CD45 (clone: HI30), CD3 (clone: UCHT1), CD56 (clone: HCD56), CD4 (clone: OKT4), CD8 (clone: RPA-T8), C–C motif chemokine receptor 8 (CCR8) (clone: L263G8), PD-1 (clone: EH12.1), Tim-3 (clone: F38-2E2), and GzmB (clone: GB11) from BioLegend (San Diego, CA, USA), and Forkhead box P3 (FOXP3) (clone: PCH101) and CD25 (clone: BC96) from Invitrogen (Waltham, MA, USA). Regarding intracellular staining, the FOXP3/Transcription Factor Staining Buffer Kit (Thermo Fisher Scientific, Waltham, MA, USA) was used. Stained cells were analyzed using a NovoCyte Quanteon Flow Cytometer (Agilent, Santa Clara, CA, USA).

### Statistical analysis

Quantitative data that did not follow a normal distribution were analyzed using the two-tailed non-parametric Mann–Whitney U test. The two-tailed Fisher’s exact probability test was used in the bivariate analysis. Cumulative survival rates were plotted using the Kaplan–Meier method, and differences were compared using the Log-rank test. A p-value < 0.05 was considered to be significant. Statistical analyses were performed using JMP Pro, version 17.0.0 (JMP, Tokyo, Japan).

### Literature search

We systematically searched PubMed for studies published between January 2000 and August 2025. The keywords used were “oral squamous cell carcinoma”, “microbiota”, “tumor-infiltrating immune cells”, and “bacterial composition”. Only original articles and reviews reporting in detail on the OSCC microbiome or tumor immunity were included.

## Results

### Patient backgrounds

Table 1 summarizes patient backgrounds in the discovery cohort. Among patients, 43% were male and cancer types were as follows: tongue cancer (22%), mandibular gingival cancer (35%), maxillary gingival cancer (26%), and buccal mucosa cancer (17%). The median number of teeth was 25. Half of the patients were classified as pT4, 30% were N + , and 50% were pathological stage (pStage) IV, indicating that 50% of this cohort had advanced cancer.

**Table 1:** Clinicopathological factors in Groups A and B in the discovery cohort

		Total (n = 23)	A (n = 13)	B (n = 10)	*P* value (A vs B)
Age, years	Median (range)	74 (43–91)	70 (46–91)	85 (43–89)	0.16
Sex, n (%)	Male	10 (43.5)	7 (30.4)	3 (13.0)	0.25
	Female	13 (56.5)	6 (26.1)	7 (30.4)	
Smoking status, n (%)	Yes	2 (8.7)	2 (8.7)	0 (0)	0.12
	No	21 (91.3)	11 (47.8)	10 (43.5)	
Drinking status, n (%)	Yes	13 (56.5)	7 (30.4)	3 (13.0)	0.25
	No	10 (43.5)	6 (26.1)	7 (30.4)	
Location, n (%)	Tongue	5 (21.7)	1 (4.3)	4 (17.4)	0.18
	Mandibular gingiva	8 (34.8)	5 (21.7)	3 (13.0)	
	Maxilla gingiva	6 (26.1)	5 (21.7)	1 (4.3)	
	Buccal mucosa	4 (17.4)	2 (8.7)	2 (8.7)	
Number of teeth, n (%)	Median (range)	25 (0–31)	26 (0–31)	16 (0–29)	0.06
Periodontal disease, n (%)	Yes	9 (39.1)	5 (21.7)	4 (17.4)	0.94
	No	14 (60.9)	8 (34.8)	6 (26.1)	
pT, n (%)	T1	3 (13.0)	1 (4.3)	2 (8.7)	0.002
	T2	8 (34.8)	1 (4.3)	7 (30.4)	
	T3	1 (4.3)	1 (4.3)	0 (0)	
	T4a	11 (47.8)	10 (43.5)	1 (4.3)	
	T4b	0 (0)	0 (0)	0 (0)	
pN, n (%)	0	16 (69.6)	7 (30.4)	9 (39.1)	0.08
	1	2 (8.7)	1 (4.3)	1 (4.3)	
	2	3 (13.0)	3 (13.0)	0 (0)	
	3	2 (8.7)	2 (8.7)	0 (0)	
pStage, n (%)	I	2 (8.7)	0 (0)	2 (8.7)	0.0003
	II	8 (34.8)	1 (4.3)	7 (30.4)	
	III	0 (0)	0 (0)	0 (0)	
	IVA	11 (47.8)	10 (43.5)	1 (4.3)	
	IVB	2 (8.7)	2 (8.7)	0 (0)	
YK classification, n (%)	2	6 (26.1)	4 (17.4)	2 (8.7)	0.59
	3	11 (47.8)	5 (21.7)	6 (26.1)	
	4C	6 (26.1)	4 (17.4)	2 (8.7)	
	4D	0 (0)	0 (0)	0 (0)	
DOI (mm), n (%)	< 10	12 (52.2)	3 (13.0)	9 (39.1)	0.0002
	≥ 10	11 (47.8)	10 (43.5)	1 (4.3)	

*pT* pathological T category, *pN* pathological N category, *pStage* pathological Stage, *YK* Yamamoto-Kohama classification, *DOI* Depth of invasion

### Microbial diversity

We compared bacterial diversity across Ts, Td, Ns, and stool samples. α-Diversity, evaluated using the Shannon index, was significantly higher in Ts and Td samples than in Ns samples (Fig. 1b). α-Diversity was also significantly higher in stool samples than in Ns samples, but did not significantly differ between Ts, Td, and stool samples. A principal co-ordinates analysis of the unweighted UniFrac distance showed that the bacterial composition of stool samples markedly differed from those of Ts, Td, and Ns samples (Fig. 1c). Furthermore, within Ts, Td, and Ns samples, the bacterial composition was not clustered based on the sampling site, it was more likely to be clustered by individual patients, indicating greater similarities in bacterial communities in Ts, Td, and Ns samples obtained from the same individual (Fig. 1d). Therefore, we mainly analyzed Ts samples using the same collection method as Ns samples.

### Classification of the bacterial composition in Ts samples

**Fig. 2:**
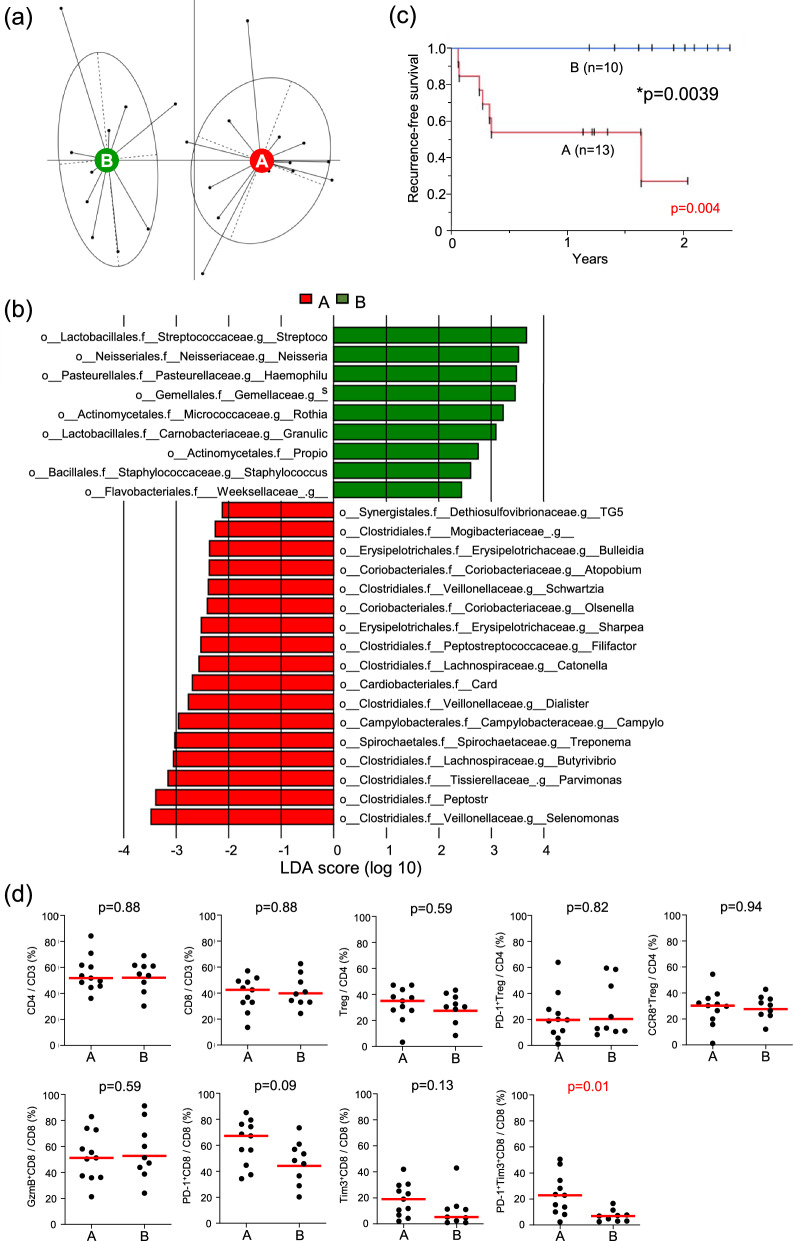
Cancer classification by the tumor surface microbiota and its link to prognosis and the tumor immune microenvironment. **a** An entero-type analysis classifying all 23 patients into two groups (Groups A and B) based on the composition of the microbiota at the genus level from the tumor surface. Each point represents one patient, with points connected by lines indicating that they belong to the same group. **b** A LEfSe analysis comparing the composition of the microbiota at the genus level between Groups A and B. Genera with a linear discriminant analysis (LDA) score > 2 are shown. **c** Recurrence-free survival (RFS) in Groups A and B. Survival rates were compared using the Log-rank test. **d** The functional status of tumor-infiltrating lymphocytes was compared between Groups A and B using flow cytometry, detecting the expression of CD3, CD4, CD8, FOXP3, PD-1, CCR8, GzmB, and Tim-3. PD-1, CCR8, Tim-3, and GzmB on CD8^+^ and CD4^+^ T cells were analyzed as exhaustion and activation markers, respectively. In **d**, horizontal lines indicate medians

We classified the bacterial composition in Ts samples based on its similarity and examined its relationships with clinical and pathological factors. An entero-type analysis was conducted on the bacterial data of Ts samples at the genus level (level-6), resulting in the classification of 23 patients into two groups based on bacterial similarity: Group A (n = 13) and Group B (n = 10) (Fig. 2a). Using a linear discriminant analysis effect size (LEfSe) analysis to compare the bacterial compositions of Groups A and B, anaerobic bacteria, such as *Selenomonas*, *Peptostreptococcus*, and *Parvimonas*, were predominantly detected in Group A, while aerobic bacteria, including *Streptococcus* and *Neisseria*, were predominantly detected in Group B (Fig. 2b).

An examination of relationships with clinical and pathological factors revealed that Group A had a significantly higher pathological T category (pT) (*p* = 0.002), depth of invasion (DOI) (*p* = 0.0002), and pStage (*p* = 0.0003) than Group B (Table 1). Regarding blood markers, Group A showed a significantly higher white blood cell count, neutrophil count, and neutrophil–lymphocyte ratio than Group B (Supplementary Table 1). Group A also had significantly shorter RFS than Group B (*p* = 0.004) (Fig. 2c). No recurrence was detected in Group B during the follow-up period. These results suggest that Group A, characterized by a predominance of anaerobic bacteria, exhibited advanced stages of cancer, a lower nutritional immune status, and a poorer prognosis.

### Relationship between the Ts bacterial composition and tumor immune profile

To investigate the relationship between the bacterial composition in Ts samples and the local immune environment of the tumor, immune cells were extracted from resected tumor specimens and TILs were analyzed by flow cytometry. The percentages of CD8^+^ and CD4^+^ T cells among CD3^+^ T cells were analyzed, along with the expression of PD-1, Tim-3, CCR8, and GzmB on each T cell (Fig. 2d). Although no significant differences were observed in the percentages of CD8^+^ or CD4^+^ T cells among CD3^+^ T cells between Groups A and B, the frequency of PD-1^+^Tim3^+^ expression in CD8^+^ T cells was significantly higher in Group A than in Group B (p = 0.01). No significant differences were noted in the other factors examined. Since PD-1^+^Tim3^+^CD8^+^ T cells are considered to be terminally exhausted T cells, Group A was considered to have a higher percentage of exhausted CD8^+^ T cells.

### Comparison of bacterial compositions across sample types and evaluation of the anaerobic bacterial score (PPS/S ratio)

**Fig. 3:**
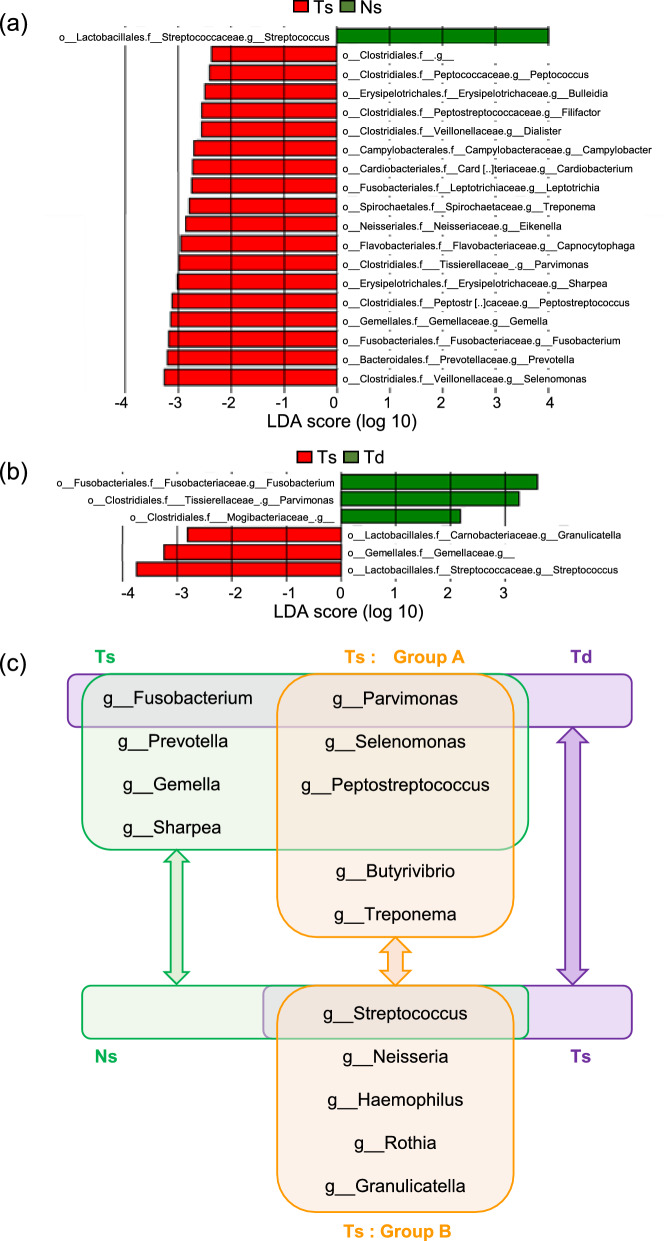
LEfSe analysis and overlap in the composition of the microbiota between samples. **a** A LEfSe analysis comparing the composition of the microbiota at the genus level between the tumor surface and normal mucosal surface. Genera with a linear discriminant analysis (LDA) score > 2 are shown. **b** A LEfSe analysis comparing the composition of the microbiota at the genus level between the tumor surface and deep in the tumor. Genera with a linear discriminant analysis (LDA) score > 2 are shown. **c** Overlap in the composition of the microbiota between samples. Common bacterial genera identified in comparisons of the microbiota between Groups A and B on the tumor surface, between the tumor surface and normal mucosal surface, and between the tumor surface and deep in the tumor are shown

**Fig. 4:**
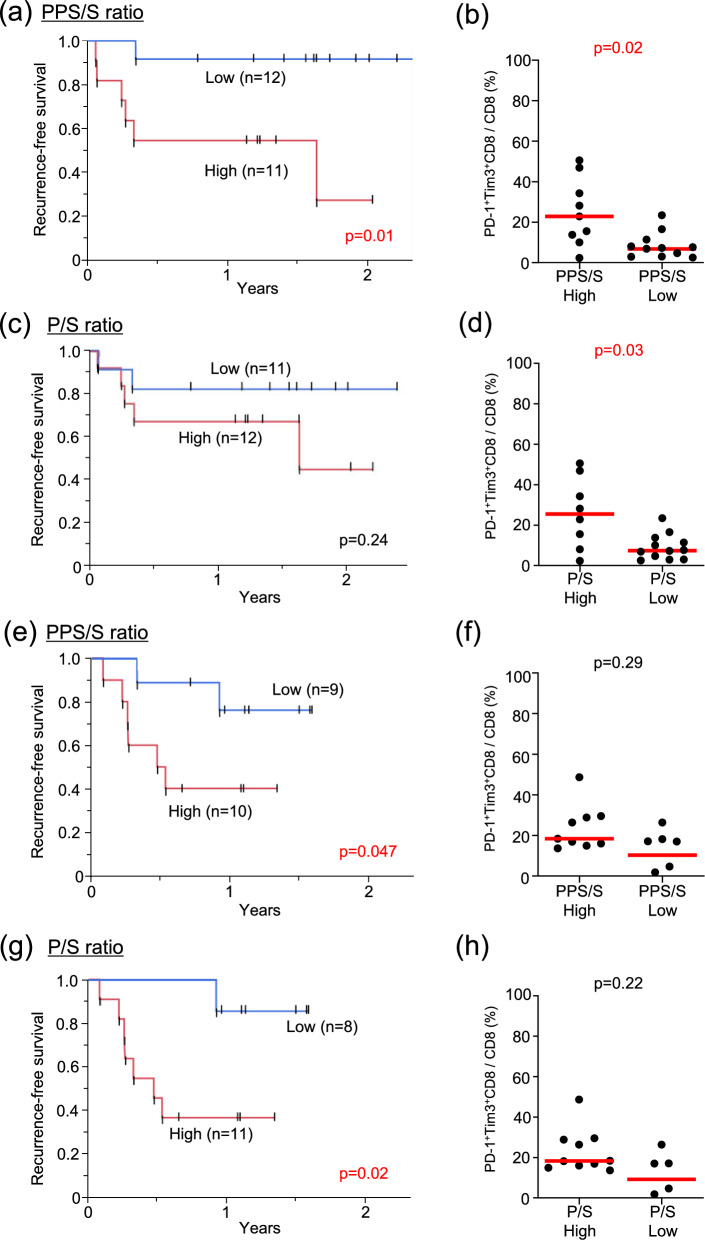
Survival and the status of tumor-infiltrating immune cells based on PPS/S and P/S ratios. **a** Recurrence-free survival (RFS) between the high and low PPS/S ratio groups in the discovery cohort. **b** The frequency of PD-1^+^Tim-3^+^ cells among CD8^+^ T cells in tumors was compared between the high and low PPS/S ratio groups in the discovery cohort. **c** RFS between the high and low P/S ratio groups in the discovery cohort. **d** The frequency of PD-1^+^Tim-3^+^ cells among CD8^+^ T cells in tumors was compared between the high and low P/S ratio groups in the discovery cohort. **e** RFS between the high and low PPS/S ratio groups in the validation cohort. **f** The frequency of PD-1^+^Tim-3^+^ cells among CD8^+^ T cells in tumors was compared between the high and low PPS/S ratio groups in the validation cohort. **g** RFS between the high and low P/S ratio groups in the validation cohort. Survival rates were compared using the Log-rank test. **h** The frequency of PD-1^+^Tim-3^+^ cells among CD8^+^ T cells in tumors was compared between the high and low P/S ratio groups in the validation cohort. In **b**, **d**, **f**, **h**, horizontal lines indicate medians

To examine differences in bacterial compositions between Ts and Ns samples as well as between Ts and Td samples, LEfSe analyses of Ts and Ns samples and of Ts and Td samples were performed. In the comparison between Ts and Ns samples, *Selenomonas*, *Prevotella*, *Fusobacterium*, *Gemella*, *Peptostreptococcus*, *Sharpea*, and *Parvimonas* were predominantly detected in Ts samples, while *Streptococcus* was predominantly detected in Ns samples (Fig. 3a). In the comparison between Ts and Td samples, *Streptococcus* was more prevalent in Ts samples, whereas *Fusobacterium* and *Parvimonas* were more prevalent in Td samples (Fig. 3b). Since several bacterial species were consistently detected across each comparison, the results obtained were consolidated into a single figure (Fig. 3c). The four bacterial genera, *Parvimonas, Peptostreptococcus*, *Selenomonas*, and *Streptococcus*, were commonly detected in comparisons between Groups A and B using Ts samples and also between Ts and Ns samples. These four bacterial species collectively accounted for 30.1% of the total bacterial composition (range: 10.8–50.2%). Importantly, these four bacterial genera were uniquely detected in Ts samples and did not overlap with stool samples (Supplementary Fig. S2). Furthermore, a differentially abundant microbiota was not identified between Groups A and B in stool Ts samples. Collectively, these results demonstrated that the bacterial communities in tumors and stools were fundamentally distinct. Regarding patient backgrounds, patients with a higher prevalence of anaerobic bacteria, such as *Parvimonas*, *Peptostreptococcus*, and *Selenomonas*, had more advanced cancer stages, shorter RFS, and a higher frequency of PD-1^+^Tim3^+^ expression in CD8^+^ T cells (Supplementary Tables S2-4, Supplementary Fig. S1a–c, S3a–c). Conversely, patients with a higher prevalence of aerobic bacteria, such as *Streptococcus*, had earlier stage cancers and longer RFS (Supplementary Table S5, Supplementary Figs. S1d, S3d). Using these four bacterial genera, we created a novel scoring system, the PPS/S ratio, calculated as (*Parvimonas* + *Peptostreptococcus* + *Selenomonas*) / (*Streptococcus*). When patients were categorized into high (n = 11) and low (n = 12) groups of the PPS/S ratio based on the median value, the high PPS/S ratio group had a significantly higher pT (*p* = 0.002), pathological N category (pN) (*p* = 0.01), pStage (*p* = 0.0001), DOI (*p* = 0.0002), and shorter RFS (*p* = 0.01) than the low PPS/S ratio group (Table 2, Fig. 4a). Furthermore, the frequency of PD-1^+^Tim3^+^ expression in CD8^+^ T cells was significantly higher in the high PPS/S ratio group than in the low PPS/S ratio group, indicating an impaired immune status in the TME of the high PPS/S ratio group (*p* = 0.02) (Fig. 4b, Supplementary Fig. S4). Therefore, the anaerobic bacterial score represented by the PPS/S ratio suggests an exhausted status of intratumoral immune cells and a poor prognosis. Notably, even when the analysis was restricted to patients with advanced-stage OSCC, a high PPS/S ratio correlated with a poor prognosis (p = 0.007) (Supplementary Fig. S5). This result suggests that the prognostic impact of the anaerobic bacterial score is not merely a reflection of the disease stage, but rather represents an independent microbiota-related factor.

**Table 2:** Clinicopathological factors in high and low PPS/S ratio groups in the discovery cohort

		PPS/S high (n = 11)	PPS/S low (n = 12)	*P* value
pT, n (%)	T1	1 (9.1)	2 (16.7)	0.0002
	T2	0 (0)	8 (66.7)	
	T3	1 (9.1)	0 (0)	
	T4a	9 (81.8)	2 (16.7)	
pN, n (%)	0	5 (45.5)	11 (91.7)	0.01
	1	1 (9.1)	1 (8.3)	
	2	3 (27.3)	0 (0)	
	3	2 (18.2)	0 (0)	
pStage, n (%)	I	0 (0)	2 (16.7)	0.0001
	II	0 (0)	8 (66.7)	
	III	0 (0)	0 (0)	
	IVA	9 (81.8)	2 (16.7)	
	IVB	2 (18.2)	0 (0)	
YK classification, n (%)	2	4 (36.4)	2 (16.7)	0.54
	3	4 (36.4)	7 (58.3)	
	4C	3 (27.3)	3 (25.0)	
	4D	0 (0)	0 (0)	
DOI (mm), n (%)	< 10	1 (9.1)	10 (83.3)	0.0002
	≥ 10	10 (90.9)	2 (16.7)	

*pT* pathological T category, *pN* pathological N category, *pStage* pathological Stage, *YK* Yamamoto-Kohama classification, *DOI* Depth of invasion

### Development of a simplified anaerobic bacterial score (P/S ratio)

Only two species were predominantly and consistently identified across comparisons of Groups A and B using Ts samples, Ts and Ns samples, and Ts and Td samples (Fig. 3c). Therefore, we further considered a simplified score with the P/S ratio, with the frequency of these two bacterial genera calculated as (*Parvimonas* / *Streptococcus*). These two species accounted for 22.8% of the total bacterial composition (range: 8.6–50.1%). After categorizing patients into high (n = 12) and low (n = 11) P/S ratio groups based on the median value, the high P/S ratio group had a significantly higher pT (*p* = 0.001), pStage (*p* = 0.0001), and DOI (*p* = 0.001) and slightly shorter RFS (*p* = 0.24) (Fig. 4c, Supplementary Table S6). Furthermore, the frequency of PD-1^+^Tim3^+^ expression in CD8^+^ T cells was significantly higher in the high P/S ratio group than in the low P/S ratio group (*p* = 0.03) (Fig. 4d, Supplementary Fig. S6). Therefore, the simplified anaerobic bacterial scoring system based on the ratio of two genera (the P/S ratio) appeared to be associated with an exhausted tumor immune state and may be of prognostic value.

### Univariate and multivariate analyses of RFS

We performed univariate and multivariate analyses of RFS in relation to clinical factors and both the PPS/S and P/S ratios in the discovery cohort. In the univariate analysis, a high PPS/S ratio (HR 9.61, 95% CI 1.15–80.63; *p* = 0.04) and pStage (HR 6.97, 95% CI 0.82–59.34; *p* = 0.08) were associated with a poor prognosis (Supplementary Table S9). However, in the multivariate analysis, neither the PPS/S ratio nor pStage emerged as an independent adverse prognostic factor, suggesting the presence of a strong confounding effect between the two variables.

### Validation of the Significance of anaerobic bacterial scores (PPS/S and P/S ratios)

The significance of the anaerobic bacterial score (PPS/S and P/S ratio) was confirmed in the validation cohort of 19 OSCC patients (Supplementary Tables S7 and S8). When patients were categorized into the high (n = 10) and low (n = 9) PPS/S ratio groups based on the same cut-off value from the discovery cohort, RFS was significantly shorter in the high PPS/S ratio group than in the low PPS/S ratio group (*p* = 0.047) (Fig. 4e). The frequency of PD-1^+^Tim3^+^ expression in CD8^+^ T cells was higher in the high PPS/S ratio group than in the low PPS/S ratio group (*p* = 0.29) (Fig. 4f). When patients were categorized into high (n = 11) and low (n = 8) P/S ratio groups using the same cut-off value from the discovery cohort, RFS was significantly shorter in the high P/S ratio group (*p* = 0.02) (Fig. 4g). The frequency of PD-1^+^Tim3^+^ expression in CD8^+^ T cells was higher in the high P/S ratio group than in the low P/S ratio group (*p* = 0.22) (Fig. 4h). The multivariate analysis identified the PPP/S ratio (HR 12.4, 95% CI 1.93–79.88; *p* = 0.008) and P/S ratio (HR 30.7, 95% CI 2.27–416.3; *p* = 0.01) as independent worse prognostic factors (Supplementary Table S10). These results confirmed the significance of the anaerobic bacterial score in the validation cohort.

## Discussion

In the present study, we comprehensively analyzed the bacterial compositions of various samples from OSCC patients and found that patients may be categorized into two groups based on the tumor surface microbiota using an entero-type analysis. A LEfSE analysis showed that *Parvimonas*, *Selenomonas*, and *Peptostreptococcus* were predominant in anaerobic bacteria-dominant Group A, while *Streptococcus* was predominant in aerobic bacteria-dominant Group B. An anaerobic bacterial scoring system, represented by the PPS/S ratio calculated using the percentages of these four bacterial genera or the simplified P/S ratio, was associated with an exhausted state of intratumoral immune cells and a poor prognosis. Therefore, our OSCC-specific scoring system effectively predicted immune cell exhaustion and patient outcomes. The bacterial composition in OSCC has already been reported in numerous studies [31–33], and has been shown to change as tumors progress [34]. Our study provides several distinct improvements over previous studies in the oral cancer microbiome field [31–39]. In contrast to many studies that focused on taxonomic descriptions alone, we developed a quantitative anaerobic bacterial scoring system (PPS/S and P/S ratios) that directly correlated with patient prognosis. We demonstrated a direct relationship between intratumoral anaerobic bacterial predominance and the functional exhaustion of tumor-infiltrating CD8⁺ T cells in OSCC. Moreover, we integrated Ts and Ns samples, highlighting the spatial specificity of anaerobic bacterial enrichment. Collectively, these factors clearly differentiate our study from previous research and underscore its novelty in linking OSCC-specific microbiome characteristics with functional immune outcomes. We also showed that the anaerobic bacterial environment was associated with immune cell exhaustion, which was a contributing factor to a poor prognosis. Consistent with previous findings showing that the infiltration of *Porphyromonas gingivalis* into OSCC promoted tumor proliferation by inducing immunosuppressive cells [40], the present results suggest that the broader anaerobic bacterial environment is associated with an immunosuppressive tumor state. Therefore, the modulation of these microbiomes may enhance anti-tumor immunity, offering a novel therapeutic strategy in OSCC.

As reported by Nejman et al., bacteria have been detected within tumor tissues in seven cancer types, including breast, lung, ovarian, pancreatic, melanoma, bone, and brain [41]. Additionally, an increasing number of studies have recently documented relationships between intratumoral bacteria and both therapeutic efficacy and prognosis [42]. Regarding the relationship between bacteria and OSCC, oral pathogenic bacteria, particularly those associated with early periodontal diseases, such as periodontitis, have been linked to the onset of malignant diseases [35]. According to epidemiological data, the incidence of OSCC in patients with periodontal disease was 57.1%, whereas it was only 28.6% in those without inflammation [36]. Bacteria that specifically promote chronic infections and the activation of inflammatory responses include *Prevotella intermedia*, *P. gingivalis*, and *Fusobacterium nucleatum*, with the latter two promoting tumorigenic transformation through different mechanisms [37]. *P. gingivalis* inhibits and modulates the host’s normal immune responses and, thus, has been implicated in the progression of OSCC [38]. Accordingly, a high relative abundance of *P. gingivalis* positively correlated with long-term survival in patients with OSCC [39]. Regarding *Fusobacterium*, a previous study demonstrated that disruption of the microbiota, with increases in the abundance of *Fusobacterium* and decreases in that of *Streptococcus*, led to the invasion of *F. nucleatum* into the mucosa because *Streptococcus* normally suppresses inflammation [43]. Infiltrating *Fusobacterium* stimulates inflammation, protects tumor cells from immune attacks, and contributes to the development of OSCC by binding to the oral epithelium via Toll-like receptors [44, 45]. 16S rDNA sequencing detected *Fusobacterium*, *Prevotella*, *Gemella*, *Sharpea*, *Parvimonas*, *Selenomonas*, and *Peptostreptococcus* in Ts samples, whereas *Streptococcus* was identified in Ns samples at the genus level. However, *P. gingivalis* and *F. nucleatum* were not differentially detected in the comparison of Groups A and B using Ts samples, which were also classified according to the degree of tumor progression. Therefore, although *P. gingivalis* and *F. nucleatum* may contribute to carcinogenesis via chronic inflammation, they do not appear to affect tumor progression.

In the present study, we established an anaerobic bacterial scoring system represented by the PPS/S ratio and demonstrated that a high PPS/S ratio correlated with a poor prognosis. The calculated percentages of the four bacterial genera accounted for 30% of the total bacterial population in Ts samples. Additionally, a high PPS/S ratio was observed in patients with advanced cancer, and even when analyses were limited to these patients, a high PPS/S ratio was associated with a poor prognosis. This result suggests that a high anaerobic bacterial score not only indicates cancer progression, but also correlates with a poor prognosis. As a bacterial biomarker, the ratio of *Firmicutes/Bacteroidetes*, analyzed at the phylum level, significantly decreased in liver cancer [46]. Moreover, a reduced ratio of *Firmicutes/Bacteroidetes* has been associated with dysbiosis in gastrointestinal tract metabolism, which results in low concentrations of circulating short-chain fatty acids and subsequently affects elements of host systemic immunity and systemic inflammation [47]. However, the scoring systems in previous studies did not predict the exhausted status of tumor-infiltrating immune cells or patient outcomes [48, 49]. In the present study, the new PPS/S ratio scoring system was developed to predict the exhausted status of tumor-infiltrating immune cells and patient outcomes. Furthermore, a simplified scoring system using the P/S ratio, based on *Parvimonas* and *Streptococcus*, indicated an exhausted status of tumor-infiltrating immune cells and a poor prognosis. The significance of the anaerobic bacterial score was confirmed in the validation cohort, indicating its potential to predict the immune status and outcomes. Moreover, since the reversal of immune exhaustion may improve anti-tumor immunity and prognosis [50], the present results indicate that controlling the intratumoral anaerobic microbiota has potential as a novel approach to reinvigorate anti-tumor immunity and improve patient outcomes.

Recent studies suggested that the modulation of the gut microbiota enhanced the efficacy of ICI; however, the underlying mechanisms remain unclear [16, 17]. One study demonstrated that gut bacteria activated intestinal dendritic cells (DCs), which subsequently stimulated tumor-associated DCs, thereby augmenting the therapeutic effects of checkpoint blockade [51]. In the present study, we showed that oral anaerobic bacteria contributed to the exhaustion of tumor-infiltrating immune cells in OSCC. These results imply that the control of oral anaerobes may restore anti-tumor immunity and mitigate immune checkpoint–mediated suppression. Since ICI are not currently covered by the national health insurance system for oral cancer, our results highlight the potential of oral bacterial control to transform existing treatment strategies. Various approaches have been examined to modulate the microbiota. Fecal microbiota transplantation, dietary interventions, probiotics, prebiotics, and engineered microbial products have been employed to reshape the intestinal microbiota and enhance anti-tumor immune responses within the TME, thereby improving the efficacy of cancer immunotherapy [52–55]. In this context, green nanomaterials may offer antimicrobial or anti-biofilm activity with tumor-targeted delivery. Silver and other biogenic nanoparticles may disrupt pathogenic oral biofilms, while gold nanoparticles provide photothermal, photodynamic, and drug-delivery functions [56–59]. The integration of these nanomaterials with microbiota-based patient selection may enable microbial control and intralesional nanomedicine to enhance cancer immunotherapy. Therefore, future studies that investigate the use of these microbiota-targeted therapies to enhance anti-tumor immunity in OSCC are needed.

In the present study, immune cells were purified from fresh tumor tissues and analyzed by flow cytometry to examine the effects of the microbiome on tumor-infiltrating immune cells. The results obtained showed that anaerobic bacteria-dominant Group A, which had a poor prognosis, had a significantly higher frequency of exhausted CD8^+^ T cells than aerobic bacteria-dominant Group B. Additionally, the high PPS/S ratio group with anaerobic bacterial dominance had a significantly higher frequency of exhausted CD8^+^ T cells than the low PPS/S ratio group with aerobic bacterial dominance. Since exhausted PD-1^+^Tim3^+^CD8^+^ T cells are unable to produce effector cytokines, such as GzmB and IFN-γ, this insufficiency may impair anti-tumor immunity. The abundant infiltration of PD-1^+^Tim3^+^CD8^+^ T cells in the TME correlated with a poor prognosis in ovarian cancer [60]. In the present study, the abundance of anaerobic bacteria correlated with exhausted PD-1^+^Tim3^+^CD8^+^ T cells in tumors, indicating the effects of anaerobic bacteria on the inactivation of tumor-infiltrating immune cells. The activation of natural immunity by specific bacteria, inflammation induced by the release of cytokines, and the activation of inhibitory mechanisms by antigen-presenting cells may be involved in this process; however, the exact mechanisms underlying the contribution of the bacterial genera detected remain unclear. Previous studies reported a causal relationship between the gut microbiota and antitumor immune responses. For example, *F. nucleatum* has been shown to inhibit NK- and T-cell activation through Fap2–TIGIT interactions [61], while *P. gingivalis* promotes tumor growth by inducing myeloid-derived suppressor cells through the secretion of IL-6 and IL-8 from oral keratinocytes [40]. Therefore, the mechanisms by which bacteria affect local immune cells need to be examined in future studies.

This study has several limitations that need to be addressed. The cohort consisted of 42 patients from a single institution, highlighting the need for further investigations on larger, multi-center cohorts. Since we did not conduct a detailed analysis of the gut microbiota or tumor immunity in vitro or in vivo, we need to examine the causal relationship between the gut microbiota and antitumor immune responses in the future. Future in vivo studies need to assess bacterial modulation and tumor growth using antibiotics or bacterial administration in OSCC mouse models, while clinical trials are warranted to investigate oral hygiene interventions that reduce anaerobic bacteria, such as tooth brushing, probiotic administration, or antibacterial mouthwash, as potential strategies to improve outcomes. Furthermore, the detection and identification of bacteria were performed using 16S rDNA sequencing, which may not fully capture the functional complexity of the microbiome. Since a shotgun metagenomic analysis allows for functional assessments and more detailed classification at the species level, it needs to be incorporated in future studies. Moreover, environmental factors, such as diet, oral hygiene, long-term antibiotic usage, and oral infections, have been shown to affect the oral microbiota [62], but were not assessed in the present study. Therefore, future studies need to consider these factors and collect appropriate data in order to properly account for their potential effects.

## Conclusion

We identified an OSCC-associated microbiome and our anaerobic bacterial scoring system may predict immune cell exhaustion and patient outcomes. The modulation of this microbiome, particularly anaerobes, may enhance anti-tumor immunity and offer a novel therapeutic strategy for OSCC.

## Supplementary Information

Supplementary Material 1.

Supplementary Material 2.

## Data Availability

The datasets used and/or analyzed during the present study are available from the corresponding author upon reasonable request.

## Abbreviations

SCC: Squamous cell carcinoma
TME: Tumor microenvironment
OSCC: Oral squamous cell carcinoma
TIL: Tumor-infiltrating lymphocyte
IHC: Immunohistochemistry
Tcf-1: T cell factor 1
TOX: Thymocyte selection-associated high mobility group box
PD-1: Programmed death-1
Tim3: T-cell immunoglobulin and mucin-domain containing-3
GzmB: Granzyme B
ICI: Immune checkpoint inhibitor
Treg: Regulatory T cell
FOXP3: Forkhead box P3
OS: Overall survival
RFS: Recurrence-free survival
CCR8: C–C motif chemokine receptor 8
LEfSe: Linear discriminant analysis effect size
pT: Pathological T category
pN: Pathological N category
DOI: Depth of invasion
pStage: Pathological stage
OTU: Operational taxonomic unit
