# Supplementary material for: Impact of oral anaerobic bacteria on the tumor immune microenvironment and prognosis of oral cancer
Source: J Transl Med. 2025 Nov 12;23:1267. doi: 10.1186/s12967-025-07189-5 (PMC12613598; doi:10.1186/s12967-025-07189-5)
Supplement: Supplementary file 1 — Supplementary Material 1. [file 12967_2025_7189_MOESM1_ESM.pdf]

Supplementary Fig.S1

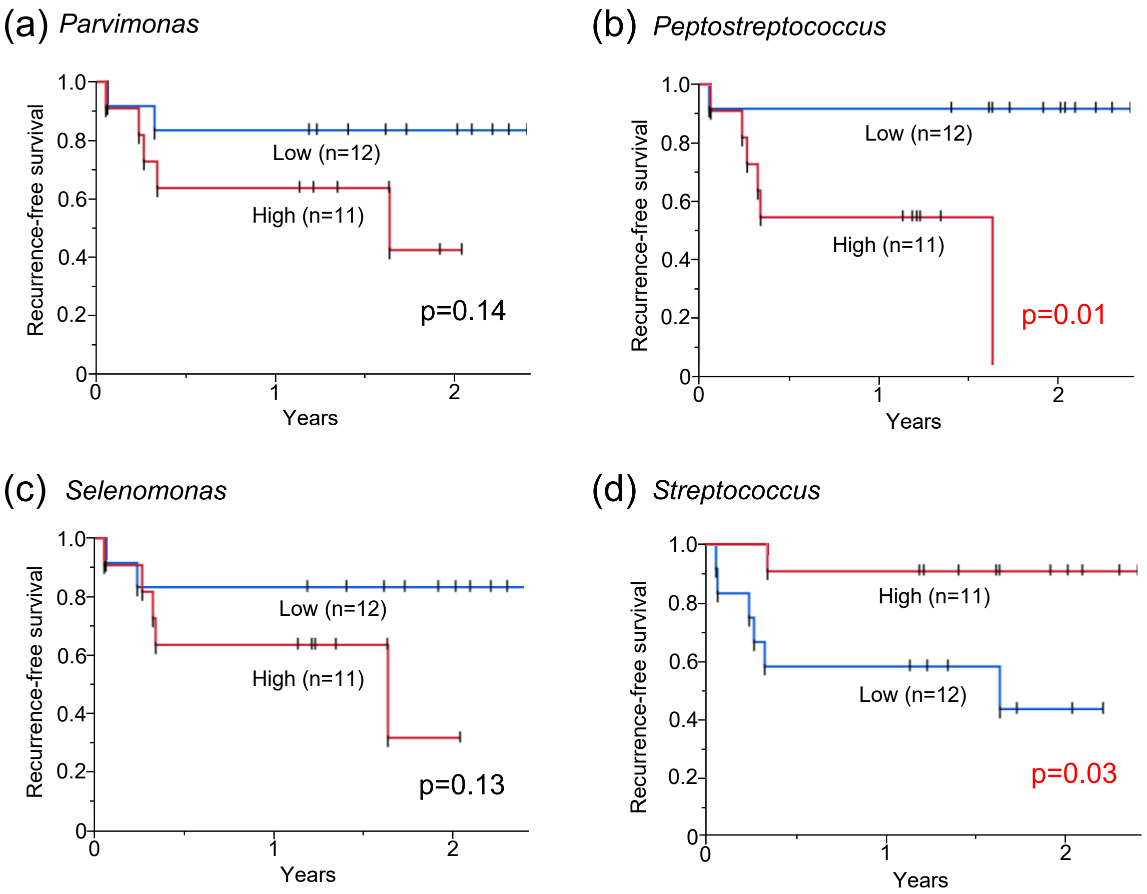

Supplementary Fig.S1. Kaplan–Meier curves comparing survival between high and low groups of each microbiota

Recurrence-free survival (RFS) between high and low groups of *Parvimonas* (A), *Peptostreptococcus* (B), *Selenomonas* (C), and *Streptococcus* (D) on the tumor surface. Survival rates were compared using the Log-rank test.

Supplementary Fig.S2

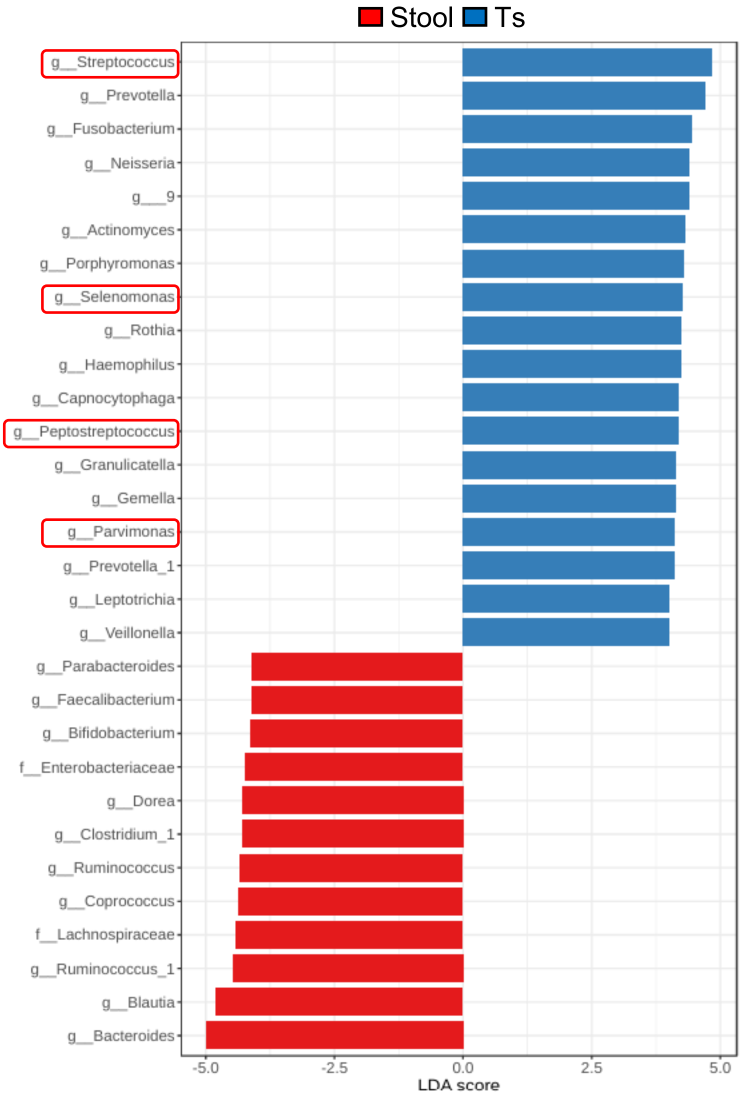

Supplementary Fig.S2. A LEfSe analysis comparing microbiota compositions between Ts and Stool
A LEfSe analysis comparing microbiota compositions at the genus level between Ts and Stool. Genera with a linear discriminant analysis (LDA) score >2 are shown.

Supplementary Fig.S3

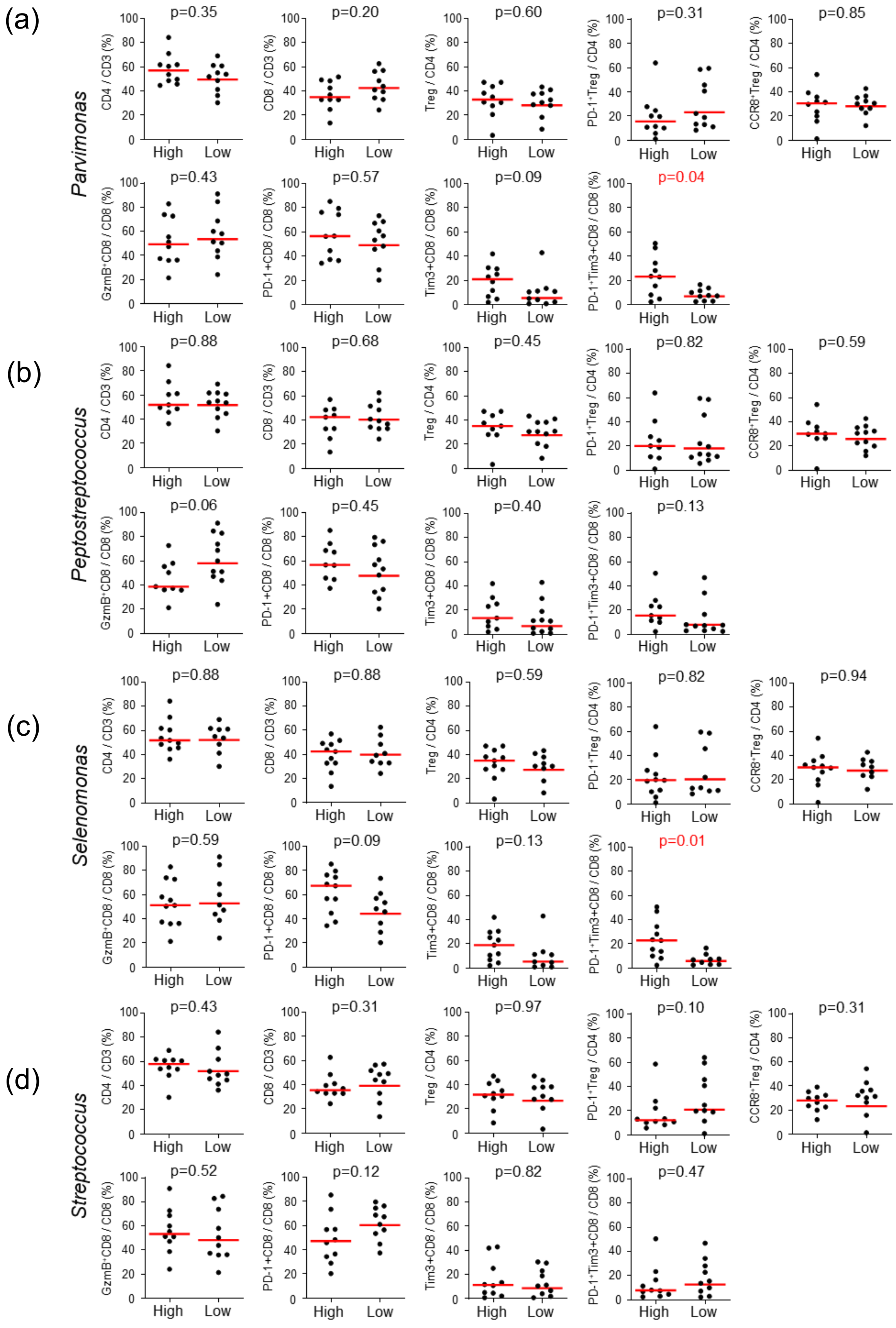

Supplementary Fig.S3. Comparison of the exhausted status of tumor-infiltrating immune cells based on high and low groups of the tumor surface microbiota.

The exhausted status of tumor-infiltrating lymphocytes was compared between the high and low groups of *Parvimonas* (A), *Peptostreptococcus* (B), *Selenomonas* (C), and *Streptococcus* (D) on the tumor surface. The exhausted status of tumor-infiltrating lymphocytes was investigated with flow cytometry, detecting the expression of CD3, CD4, CD8, FOXP3, PD-1, CCR8, GzmB, and Tim-3. PD-1, CCR8, Tim-3, and GzmB on $CD8^+$ and $CD4^+$ T cells were analyzed as exhaustion and activation markers, respectively. Horizontal lines indicate medians.

Supplementary Fig.S4

PPS/S ratio

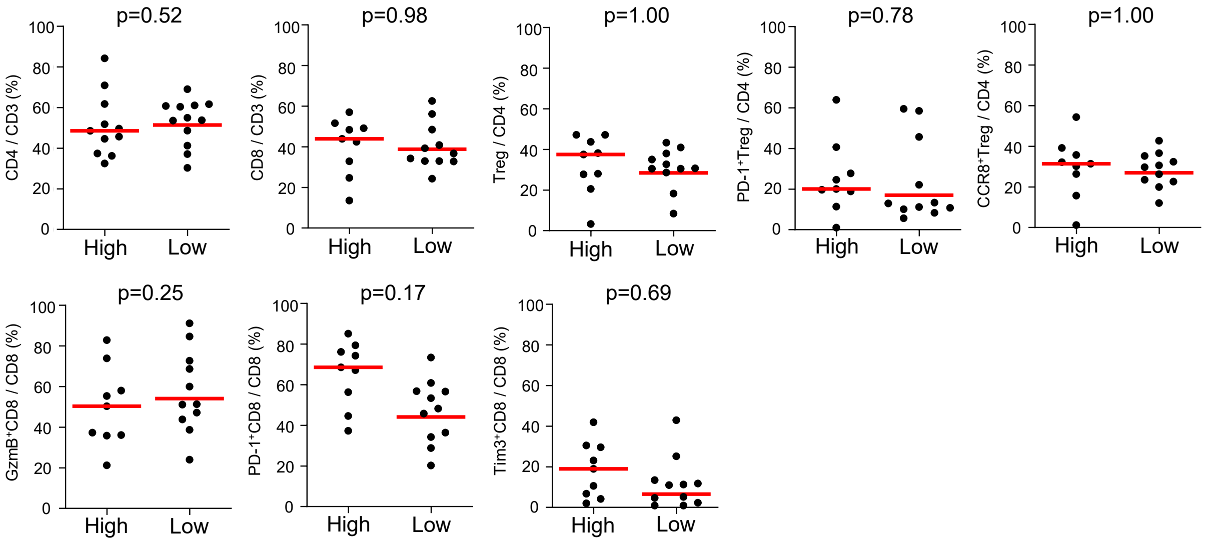

Supplementary Fig.S4. Comparison of the exhausted status of tumor-infiltrating immune cells based on the PPS/S Ratio

The exhausted status of tumor-infiltrating lymphocytes was compared between the high and low groups of the PPS/S ratio. Horizontal lines indicate medians.

Supplementary Fig.S5

PPS/S ratio

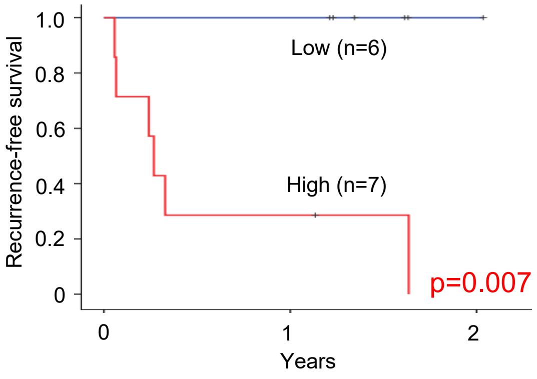

Supplementary Fig.S5. Kaplan–Meier curves comparing survival between high and low groups of the PPS/S ratio in advanced-stage OSCC

Recurrence-free survival (RFS) between high and low groups of the PPS/S ratio in advanced-stage OSCC . The cutoff value for the PPP/S ratio was set at the median. Survival rates were compared using the Log-rank test.

Supplementary Fig.S6

P/S ratio

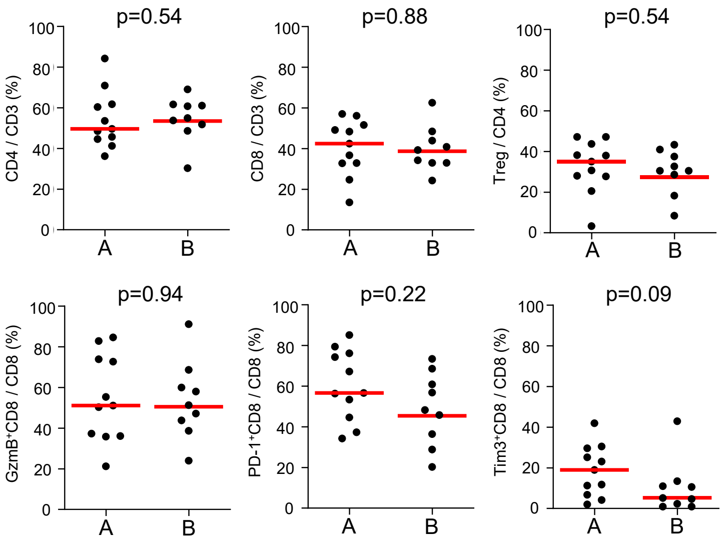

Supplementary Fig.S6. Comparison of the exhausted status of tumor-infiltrating immune cells based on the P/S ratio

The exhausted status of tumor-infiltrating lymphocytes was compared between the high and low groups of the P/S ratio. Horizontal lines indicate medians.
