# Supplementary material for: Impact of oral anaerobic bacteria on the tumor immune microenvironment and prognosis of oral cancer
Source: J Transl Med. 2025 Nov 12;23:1267. doi: 10.1186/s12967-025-07189-5 (PMC12613598; doi:10.1186/s12967-025-07189-5)
Supplement: Supplementary file 2 — Supplementary Material 2. [file 12967_2025_7189_MOESM2_ESM.pdf]

Supplementary Table S1. Blood test markers in patients in Groups A and B in the discovery cohort

	A (n=13)	B (n=10)	P value
WBC (10×)	7.2 (2.3-12)	5.3 (4.4-7.5)	0.008
Hemoglobin (g/dl)	13.2 (10.6-15.6)	13.2 (10.1-15.0)	0.88
Neutrophils (%)	70.2 (51.2-79.9)	61.3 (56.7-68.4)	0.004
Lymphocytes (%)	18.9 (11.5-35.3)	25.9 (19.9-35.4)	0.004
NLR	3.6 (1.5-6.8)	2.4 (1.6-3.4)	0.005
CRP (mg/d)	0 (0-6.4)	0 (0-2.4)	0.18
Alb (g/dl)	4.1 (3.2-4.7)	4.0 (3.7-4.8)	0.71

WBC: White blood cell, NLR: Neutrophil-to-lymphocyte ratio, CRP: C-reactive protein, Alb: Albumin

Supplementary Table S2. Clinicopathological factors in high and low groups of *Parvimonas* in the discovery cohort

		Parvimonas high (n=11)	*Parvimonas* low (n=12)	P value
pT, n (%)	T1	2 (8.7)	1 (4.3)	0.06
	T2	1 (4.3)	7 (30.4)	
	T3	1 (4.3)	0 (0)	
	T4a	7 (30.4)	4 (17.4)	
pN, n (%)	0	7 (30.4)	9 (39.1)	0.36
	1	1 (4.3)	1 (4.3)	
	2	2 (8.7)	1 (4.3)	
	3	1 (4.3)	1 (4.3)	
pStage, n (%)	I	1 (4.3)	1 (4.3)	0.06
	II	1 (4.3)	7 (30.4)	
	III	0 (0)	0 (0)	
	IVA	8 (34.8)	3 (13.0)	
	IVB	1 (4.3)	1 (4.3)	
YK classification, n (%)	2	3 (13.0)	3 (13.0)	0.97
	3	5 (21.7)	6 (26.1)	
	4C	3 (13.0)	3 (13.0)	
	4D	0 (0)	0 (0)	
DOI (mm), n (%)	<10	3 (13.0)	8 (34.8)	0.06
	≥10	8 (34.8)	4 (17.4)	

pT: Pathological T category, pN: Pathological N category, pStage: Pathological Stage, YK: Yamamoto-Kohama classification, DOI: Depth of invasion

Supplementary Table S3. Clinicopathological factors in high and low groups of *Peptostreptococcus* in the discovery cohort

		Peptostreptococcus high (n=11)	*Peptostreptococcus* low (n=12)	P value
pT, n (%)	T1	0 (0)	3 (13.0)	0.02
	T2	2 (8.7)	6 (26.1)	
	T3	1 (4.3)	0 (0)	
	T4a	8 (34.8)	3 (13.0)	
pN, n (%)	0	6 (26.1)	10 (43.5)	0.27
	1	1 (4.3)	1 (4.3)	
	2	3 (13.0)	0 (0)	
	3	1 (4.3)	1 (4.3)	
pStage, n (%)	I	0 (0)	2 (8.7)	0.07
	II	2 (8.7)	6 (26.1)	
	III	0 (0)	0 (0)	
	IVA	8 (34.8)	3 (13.0)	
	IVB	1 (4.3)	1 (4.3)	
YK classification, n (%)	2	4 (17.4)	2 (8.7)	0.48
	3	4 (17.4)	7 (30.4)	
	4C	2 (8.7)	4 (17.4)	
	4D	0 (0)	0 (0)	
DOI (mm), n (%)	<10	2 (8.7)	9 (39.1)	0.005
	≥10	9 (39.1)	3 (13.0)	

pT: Pathological T category, pN: Pathological N category, pStage: Pathological Stage, YK: Yamamoto-Kohama classification, DOI: Depth of invasion

Supplementary Table S4. Clinicopathological factors in high and low groups of *Selenomonas* in the discovery cohort

		Selenomonas high (n=11)	*Selenomonas* low (n=12)	P value
pT, n (%)	T1	1 (4.3)	2 (8.7)	0.03
	T2	1 (4.3)	7 (30.4)	
	T3	1 (4.3)	0 (0)	
	T4a	8 (34.8)	3 (13.0)	
pN, n (%)	0	6 (26.1)	10 (43.5)	0.27
	1	1 (4.3)	1 (4.3)	
	2	3 (13.0)	0 (0)	
	3	1 (4.3)	1 (4.3)	
pStage, n (%)	I	0 (0)	2 (8.7)	0.006
	II	1 (4.3)	7 (30.4)	
	III	0 (0)	0 (0)	
	IVA	9 (39.1)	2 (8.7)	
	IVB	1 (4.3)	1 (4.3)	
YK classification, n (%)	2	2 (8.7)	4 (17.4)	0.49
	3	5 (21.7)	6 (26.1)	
	4C	4 (17.4)	2 (8.7)	
	4D	0 (0)	0 (0)	
DOI (mm), n (%)	<10	2 (8.7)	9 (39.1)	0.005
	≥10	9 (39.1)	3 (13.0)	

pT: Pathological T category, pN: Pathological N category, pStage: Pathological Stage, YK: Yamamoto-Kohama classification, DOI: Depth of invasion

Supplementary Table S5. Clinicopathological factors in high and low groups of *Streptococcus* in the discovery cohort

		Streptococcus high (n=11)	*Streptococcus* low (n=12)	P value
pT, n (%)	T1	2 (8.7)	1 (4.3)	0.11
	T2	6 (26.1)	2 (8.7)	
	T3	0 (0)	1 (4.3)	
	T4a	3 (13.0)	8 (34.8)	
pN, n (%)	0	9 (39.1)	7 (30.4)	0.04
	1	2 (8.7)	0 (0)	
	2	0 (0)	3 (13.0)	
	3	0 (0)	2 (8.7)	
pStage, n (%)	I	2 (8.7)	0 (0)	0.02
	II	6 (26.1)	2 (8.7)	
	III	0 (0)	0 (0)	
	IVA	3 (13.0)	8 (34.8)	
	IVB	0 (0)	2 (8.7)	
YK classification, n (%)	2	1 (4.3)	5 (21.7)	0.05
	3	8 (34.8)	3 (13.0)	
	4C	2 (8.7)	4 (17.4)	
	4D	0 (0)	0 (0)	
DOI (mm), n (%)	<10	8 (34.8)	3 (13.0)	0.02
	≥10	3 (13.0)	9 (39.1)	

pT: Pathological T category, pN: Pathological N category, pStage: Pathological Stage, YK: Yamamoto-Kohama classification, DOI: Depth of invasion

Supplementary Table S6. Clinicopathological factors in high and low P/S ratio groups in the discovery cohort

		P/S high (n=12)	P/S low (n=11)	P value
pT, n (%)	T1	1 (8.3)	2 (18.2)	0.001
	T2	2 (16.7)	6 (54.5)	
	T3	1 (8.3)	0 (0)	
	T4a	8 (66.7)	3 (27.3)	
pN, n (%)	0	7 (58.3)	9 (81.8)	0.13
	1	1 (8.3)	1 (9.1)	
	2	3 (25.0)	0 (0)	
	3	1 (8.3)	1 (9.1)	
pStage, n (%)	I	0 (0)	2 (18.2)	0.0001
	II	2 (16.7)	6 (54.5)	
	III	0 (0)	0 (0)	
	IVA	9 (75.0)	2 (18.2)	
	IVB	1 (8.3)	1 (9.1)	
YK classification, n (%)	2	4 (33.3)	2 (18.2)	0.66
	3	4 (33.3)	7 (63.6)	
	4C	4 (33.3)	2 (18.2)	
	4D	0 (0)	0 (0)	
DOI (mm), n (%)	<10	3 (25.0)	8 (72.7)	0.001
	≥10	9 (75.0)	3 (27.3)	

pT: Pathological T category, pN: Pathological N category, pStage: Pathological Stage, YK: Yamamoto-Kohama classification, DOI: Depth of invasion

Supplementary Table S7. Clinicopathological factors in high and low PPS/S ratio groups in the validation cohort

		PPS/S high (n=10)	PPS/S low (n=9)	P value
pT, n (%)	T1	0 (0)	3 (33.3)	0.15
	T2	4 (40.0)	4 (44.4)	
	T3	1 (10.0)	0 (0)	
	T4a	5 (50.0)	2 (22.2)	
pN, n (%)	0	9 (90.0)	5 (55.6)	0.25
	1	0 (0)	1 (11.1)	
	2	1 (10.0)	1 (11.1)	
	3	0 (0)	2 (22.2)	
pStage, n (%)	I	0 (0)	3 (33.3)	0.02
	II	4 (40.0)	2 (22.2)	
	III	1 (10.0)	1 (11.1)	
	IVA	5 (50.0)	0 (0)	
	IVB	0 (0)	3 (33.3)	
YK classification, n (%)	2	1 (10.0)	4 (44.4)	0.33
	3	6 (60.0)	4 (44.4)	
	4C	3 (30.0)	1 (11.1)	
	4D	0 (0)	0 (0)	
DOI (mm), n (%)	<10	8 (80.0)	7 (77.8)	0.91
	≥10	2 (20.0)	2 (22.2)	

pT: Pathological T category, pN: Pathological N category, pStage: Pathological Stage, YK: Yamamoto-Kohama classification, DOI: Depth of invasion

Supplementary Table S8. Clinicopathological factors in high and low P/S ratio groups in the validation cohort

		P/S high (n=11)	P/S low (n=8)	P value
pT, n (%)	T1	1 (9.1)	2 (25.0)	0.31
	T2	7 (36.4)	1 (12.5)	
	T3	0 (0)	1 (12.5)	
	T4a	3 (27.3)	4 (50.0)	
pN, n (%)	0	8 (72.7)	6 (75.0)	0.91
	1	1 (9.1)	0 (0)	
	2	1 (9.1)	1 (12.5)	
	3	1 (9.1)	1 (12.5)	
pStage, n (%)	I	1 (9.1)	2 (25.0)	0.46
	II	5 (45.5)	1 (12.5)	
	III	1 (9.1)	1 (12.5)	
	IVA	2 (18.2)	3 (37.5)	
	IVB	2 (18.2)	1 (12.5)	
YK classification, n (%)	2	4 (36.4)	1 (12.5)	0.72
	3	5 (45.5)	5 (62.5)	
	4C	2 (18.2)	2 (25.0)	
	4D	0 (0)	0 (0)	
DOI (mm), n (%)	<10	10 (90.9)	5 (62.5)	0.13
	≥10	1 (9.1)	3 (37.5)	

pT: Pathological T category, pN: Pathological N category, pStage: Pathological Stage, YK: Yamamoto-Kohama classification, DOI: Depth of invasion

Supplementary Table S9. Univariate and multivariate analyses of the recurrence-free interval in the discovery cohort

	Univariate analysis HR (95% CI)	*P* value	Multivariate analysis HR (95% CI)	*P* value	Multivariate analysis HR (95% CI)	*P* value
Age (Years) (≥74 vs. <74)	1.24 (0.27-5.65)	0.78				
Sex (Male vs. Female)	0.39 (0.08-2.04)	0.27				
Location (mobile vs. fixed)*	0.54 (0.10-2.80)	0.46				
Number of teeth (≥25 vs. <25)	0.63 (0.14-2.83)	0.54				
pT (3, 4a, 4b vs. 1,2)	2.90 (0.55-15.31)	0.21				
pN (1, 2, 3 vs. 0)	4.47 (0.99-20.26)	0.05				
pStage (III, IV vs. 0, I, II)	6.97 (0.82-59.34)	0.08	3379 (0.0-5.356E+129.)	0.96	7.58 (0.64-90.9)	0.11
YK classification (4C,4D vs. 2,3)	1.16 (0.22-6.02)	0.86				
DOI (mm) (≥10 vs. <10)	2.90 (0.55-15.31)	0.21				
PPP/S ratio (≥0.3 vs. <0.3)	9.61 (1.15-80.63)	0.04	28071 (0.0-4.424E+130)	0.95		
P/S ratio (≥0.1 vs. <0.1)	2.58 (0.50-13.36)	0.26			1.14 (0.17-7.63)	0.89

HR, Hazard ratio; CI, Confidence interval; pStage: Pathological Stage, YK classification: Yamamoto-Kohama classification, DOI: Depth of invasion

Variables with p<0.10 in the univariate analysis were assessed in the multivariate analysis.

*Mobile; Tongue, Buccal mucosa. Fixed; Mandibular gingiva, Maxilla gingiva.

Supplementary Table S10. Univariate and multivariate analyses of the recurrence-free interval in the validation cohort

	Univariate analysis HR (95% CI)	*P* value	Multivariate analysis HR (95% CI)	*P* value	Multivariate analysis HR (95% CI)	*P* value
Age (Years) (≥74 vs. <74)	1.92 (0.46-8.07)	0.37				
Sex (Male vs. Female)	0.33 (0.08-1.39)	0.13				
Location (mobile vs. fixed)*	0.49 (0.12-1.96)	0.31				
Number of teeth (≥25 vs. <25)	0.13 (0.02-1.10)	0.06	0.05 (0.005-0.47)	0.009	0.04 (0.003-0.48)	0.01
pT (3, 4a, 4b vs. 1,2)	1.04 (0.25-4.36)	0.96				
pN (1, 2, 3 vs. 0)	1.17 (0.23-5.85)	0.85				
pStage (III, IV vs. 0, I, II)	1.02 (0.25-4.08)	0.98				
YK classification (4C,4D vs. 2,3)	1.88 (0.38-9.42)	0.44				
DOI (mm) (≥10 vs. <10)	0.45 (0.05-3.65)	0.45				
PPP/S ratio (≥0.3 vs. <0.3)	4.06 (0.81-20.39)	0.08	12.4 (1.93-79.88)	0.008		
P/S ratio (≥0.1 vs. <0.1)	8.43 (1.02-69.72)	0.04			30.7 (2.27-416.3)	0.01

HR, Hazard ratio; CI, Confidence interval; pStage: Pathological Stage, YK classification: Yamamoto-Kohama classification, DOI: Depth of invasion

Variables with p<0.10 in the univariate analysis were assessed in the multivariate analysis.

*Mobile; Tongue, Buccal mucosa, floor of the mouth. Fixed; Mandibular gingiva, Maxilla gingiva.
